# 1314. Missed Opportunities for Early Treatment and Prevention of Community Acquired Bacterial Meningitis

**DOI:** 10.1093/ofid/ofad500.1153

**Published:** 2023-11-27

**Authors:** Glenn H Beard, Rodrigo Hasbun

**Affiliations:** McGovern School of Medicine, Houston, Texas; UT Health Mc Govern Medical School, Houston, Texas

## Abstract

**Background:**

Community acquired bacterial meningitis (CABM) is a life-threating infection with high rates of mortality and chronic neurological sequela. These rates increase significantly if antibiotic and steroid therapies are delayed. As such, taking every opportunity to quickly diagnosis and rapidly treat CABM is paramount to improving patient outcomes.

**Methods:**

To identify such opportunities we performed a multicenter retrospective analysis of patients who had been hospitalized for CABM in Houston, TX, from 5/2004 to 2/2023. Exact dates and times were collected for initial presentations, initiation of medications, and if the patient had been evaluated by a medical provider prior to hospitalization. Delayed antibiotics and steroids were defined as administration > 6 hours after presentation. Steroids given after antibiotics were also classified as delayed. Patients lacking exact dates and times of these variables were omitted.

**Results:**

100 CABM cases were reviewed and documentation showed that 36 patients (36%) were seen by a medical provider within 2 weeks prior to hospitalization. These patients’ chief complaints were most commonly ear pain, congestion, or dental pain. 12 patients received penicillins (4), fluoroquinolones (3), azithromycin (2), doxycycline (1), cephalosporin (1) and unspecified antibiotics (1). The other 24 patients were diagnosed with non-infectious causes (headaches, migraines, and muscle sprains).

Most hospitalizations had delayed antibiotics (53%) and steroids (73%). A significant number of patients did not receive steroids during their hospitalizations (21%). In total, 96% of patients missed opportunities for early interventions that improve outcomes.

**Conclusion:**

It appears that there are many opportunities to improve early recognition of CABM and treatment of infections that lead to CABM in the outpatient setting. This is especially true when considering that this study is more likely to underestimate the number of patients who seek care prior to hospitalization. Regarding inpatient opportunities, increasing use of steroids in the acute workup phase of bacterial meningitis should be pursued given the high rate of delayed administration and severe neurological complications that are more likely to occur when it is not administered.

**Disclosures:**

**Rodrigo Hasbun, MD MPH FIDSA**, BIOFIRE: Advisor/Consultant

